# Please, carefully, pass the P5C

**DOI:** 10.1093/jxb/erad446

**Published:** 2024-02-02

**Authors:** Paul E Verslues

**Affiliations:** Institute of Plant and Microbial Biology, Academia Sinica, Taipei 11528, Taiwan

**Keywords:** Metabolic channelling, mitochondria, ornithine aminotransferase, P5C dehydrogenase, proline dehydrogenase, stress response

## Abstract

This article comments on:

Zheng Y, Cabassa-Hourton C, Eubel H, Chevreux G, Lignieres L, Crilat E, Braun H-P, Lebreton S, Savouré A. 2024. Pyrroline-5-carboxylate metabolism protein complex detected in *Arabidopsis thaliana* leaf mitochondria. Journal of Experimental Botany 75, 917–934.

**Proline accumulates as a protective compatible solute to increase cellular osmolarity while also protecting cellular structure and function during abiotic stress. Conversely, catabolism of proline is associated with cell death. The difference between these two faces of proline metabolism depends largely on how Δ** ^**1**^ **-pyrroline-5-carboxylate (P5C), the common intermediate of proline synthesis and catabolism, is metabolized. Zheng *et al.* (2024) have provided evidence that key enzymes of P5C metabolism in the mitochondria interact with each other. This potential formation of enzyme complexes to facilitate metabolic channelling of P5C has implications for how proline catabolism contributes to respiration and redox balance to sustain cellular activity versus reactive oxygen species (ROS) production and cellular damage. The ability to switch between different states of proline catabolism may allow plants to respond to different types of environmental stimuli.**

Proline metabolism is involved in a surprisingly wide array of physiological responses. During abiotic stress, high levels of proline build up as a compatible solute (Verslues and Sharma, 2010). Compatible solutes are highly soluble, often zwitterionic, molecules that can accumulate to high cytoplasmic levels to help retain water and maintain turgor while also protecting protein and membrane structure. Conversely, during starvation conditions, in meristem tissues during stress, or during recovery from drought or salt stress, proline can serve as an alternative respiratory substrate (Sharma *et al.,* 2011; Launay *et al.,* 2019; Oh *et al.,* 2022). During incompatible pathogen infection, proline catabolism is required for programmed cell death around the infection site (Cecchini *et al.,* 2011; Senthil-Kumar and Mysore, 2012; Alvarez *et al.,* 2022). How can proline metabolism have such seemingly disparate functions? The key is the cycle of proline synthesis in the cytoplasm versus proline catabolism in the mitochondria (Box 1; Fig. 1). This cycle not only produces or catabolizes proline, it is also a determinant of, and responsive to, cellular redox status and ROS production (Zheng *et al.,* 2021; Verslues *et al.,* 2023).

**Fig. 1.**
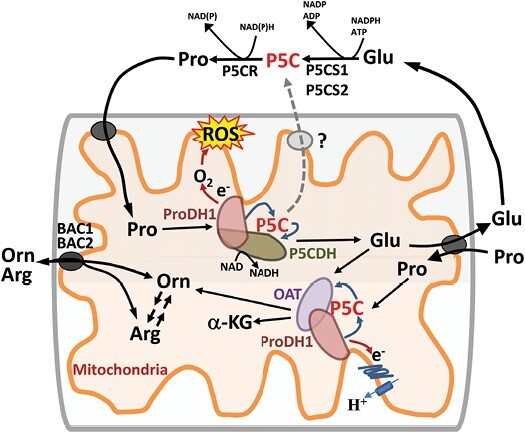
Diagram of proline metabolism and potential P5C metabolic channelling in the mitochondrial lumen. Description of the enzymes, transporters, and metabolic pathways shown can be found in Box 1.

Box 1. **Proline metabolism and possible P5C channelling in mitochondria**

Proline is synthesized by two cytosolic enzymes with Δ^1^-pyroline-5-carboxylate (P5C) as the intermediate (Fig. 1). P5C synthetase (P5CS) is rate limiting for proline synthesis, and most plants contain at least one P5CS isoform that is induced by stress and one or more other isoforms that are more essential for ‘housekeeping’ proline synthesis and development. The second step in proline synthesis catalysed by P5C reductase (P5CR) is generally not thought to be rate limiting. For catabolism, proline is translocated into mitochondria via a proline–glutamate exchanger or proline importer. The first and rate-limiting step of proline catabolism is catalysed by proline dehydrogenase (ProDH, sometimes referred to as proline oxidase). ProDH activity is typically down-regulated by abiotic stresses such as drought or salinity that lead to high levels of proline accumulation. ProDH is associated with the inner mitochondrial membrane and is relatively unique in that it can pass reductants directly into the mitochondrial electron transport. This allows proline to fuel respiratory activity. However, ProDH1 can also reduce oxygen and generate reactive oxygen species (ROS). The second step of proline catabolism is catalysed by P5C dehydrogenase (P5CDH). P5CDH can also be responsive to stress, but does not always match the response of ProDH. Ornithine aminotransferase (OAT) utilizes P5C and glutamate to produce ornithine, and thus connects proline metabolism with arginine metabolism.

While these enzymes have traditionally been thought to act independently of each other, Zheng *et al.* (2024) provide evidence that ProDH1 of *Arabidopsis thaliana* interacts with P5CDH and OAT. It is also possible that all three enzymes are present in the same large complex (not shown in Fig. 1). The formation of such complexes suggests that P5C is passed directly from ProDH to P5CDH or OAT rather than freely diffusing in the mitochondria. Extensive metabolic channelling of P5C would make sense as P5C is a potentially dangerous metabolite for two reasons. Firstly, the operation of a proline–P5C cycle, with P5C transported across the mitochondrial membrane to reach P5CR, is thought to be a way to amplify mitochondrial ROS production. Such a P5C cycle, if it exists, would need to be tightly regulated. Secondly, P5C has been proposed to have its own pro-apoptosis signalling function. The assembly of ProDH1-containing enzyme complexes may be a way to control the levels of free P5C while allowing efficient catabolism of proline for mitochondrial energy production or production of glutamate as a substrate for other metabolic pathways. Disassembly of such complexes could free P5C for other activities.

The connection of proline metabolism to redox status comes from both the synthesis and catabolism sides of proline metabolism. Proline synthesis serves as a mechanism to maintain an appropriate NADP^+^/NADPH ratio by consuming NADPH and generating NADP^+^ (Sharma *et al.,* 2011). This, along with other data, such as transcriptional profiling of *p5cs1-4*, suggests that proline synthesis is connected to photosynthesis as a safety valve for excess reductants (Shinde *et al.,* 2016; Zheng *et al.,* 2021), albeit that the exact mechanisms and localization of key enzymes and transporters mediating such a connection remain unclear. For the proline catabolism side, research in both plant and animal systems indicates that proline dehydrogenase (ProDH) can donate electrons to ubiquinone or coenzyme Q on the mitochondrial inner membrane and thus can play a major role to sustain mitochondrial electron transport or generation of ROS (Wanduragala *et al.,* 2010; Hancock *et al.,* 2016; Tanner *et al.,* 2018; Launay *et al.,* 2019). When proline catabolism is particularly high, such as during recovery from drought or salt stress when high levels of accumulated proline are recycled for other metabolic fates, the reductant generated from proline catabolism must be dissipated by alternative oxidase (Oh *et al.,* 2022). Thus, proline catabolism can not only sustain mitochondrial electron transport, but also has the capacity to overload it.

## The importance of P5C

P5C, the common intermediate of proline synthesis and catabolism, probably has its own roles in promoting cell death. For example, during pathogen infection, the combination of high P5C synthetase (P5CS) and ProDH expression with relatively low P5C dehydrogenase (P5CDH), a combination expected to increase P5C levels, is associated with programmed cell death (Qamar *et al.,* 2015). Studies in cancer biology also indicate that P5C is a pro-cell death metabolite (Phang, 2019). In both cases, it is unclear whether P5C itself has a pro-apoptosis role or whether it is associated with cell death via high levels of mitochondrial ROS generation (or both). Regardless of the underlying mechanism, data from multiple organisms indicate that while P5C is the essential intermediate of both proline synthesis and proline catabolism, it is also a potentially dangerous metabolite whose levels must be tightly controlled.

Metabolic channelling, whereby a metabolic intermediate does not freely diffuse between two enzymatic reactions but is instead passed directly from one enzyme to the next (Pareek *et al.,* 2021), is one way to tightly control the levels of a metabolic intermediate such as P5C. Enzymes that physically interact to facilitate metabolic channelling can be referred to as a metabolon (Zhang and Fernie, 2021). As discussed by Zheng *et al.* (2024), in many bacteria, the ProDH and P5CDH enzymatic activities are combined in one bifunctional protein. In other cases, the two activities are present on separate proteins, but these proteins interact, presumably to form a P5C metabolon. Given that P5C is a potentially dangerous metabolite, especially in plants that accumulate high levels of proline as a compatible solute, one obvious question is why do plants maintain separate ProDH and P5CDH proteins rather than a single bifunctional enzyme? Interaction of the enzymes to form a P5C metabolon could accomplish the same goal of sequestering P5C; however, surprisingly little is known about whether a P5C metabolon exists in plants.

## Is there P5C metabolic channelling in plants?

To begin to address this question, Zheng *et al.* (2024), used several techniques to investigate the interaction and submitochondrial localization of three P5C metabolism enzymes in *Arabidopsis thaliana*: ProDH1, P5CDH, and ornithine aminotransferase (OAT) (Box 1; Fig. 1). Bimolecular fluorescence complementation assays and pull-down experiments indicated that ProDH1 and P5CDH physically interact. As expected, ProDH1 was localized along the inner side of the mitochondrial inner membrane while P5CDH was localized both along the membrane and in the mitochondrial lumen. This suggests that while these two proteins can interact, they may not do so all the time (or not all copies of ProDH1 and P5CDH interact with each other). Similar evidence indicates that ProDH1 also associates with OAT. Native gel separation of protein complexes isolated from plants subjected to dark-induced starvation, which stimulates proline catabolism, revealed that a portion of ProDH1, P5CDH, and OAT co-migrated, consistent with their presence together in a protein complex.

As discussed in detail by Zhang and Fernie (2021), two enzymes interacting is not itself evidence of metabolic channelling. However, given the information available from other organisms, the results presented by Zheng *et al.* (2024) suggest that plants also have ways to channel and control the fate of P5C. Now that Zheng *et al.* (2024) have established key parameters for studying ProDH1–P5CDH/OAT association, it will be of interest to see whether the interaction of these proteins is altered in response to different environmental stimuli. Indeed, the likely answer to the question of why plants maintain separate ProDH and P5CDH enzymes is that the accumulation of P5C serves a purpose under certain conditions, such as the promotion of cell death during the hypersensitive response. Thus, the flexibility to assemble or disassemble a P5C metabolon may have value to plants in responding to different environmental stimuli. It will also be of interest to see how this possible P5C metabolon is compatible with other data showing that the N-terminus of ProDH1 is required for oligomerization and enzymatic activity (Fabro *et al.,* 2020). The portion of the ProDH1 protein required for interaction with P5CDH and OAT remains to be determined, thus it is unclear whether the formation of ProDH1–P5CDH/OAT complexes is in competition with assembly of ProDH1 oligomers or whether the two process happen at the same time.

## The elusive P5C cycle and other mysteries of proline catabolism

The results by Zheng *et al.* (2024) give new impetus to investigate another recurring, yet unproven, hypothesis about plant proline metabolism: the existence of a P5C cycle. It has been proposed that P5C produced by ProDH can be directly used by P5C reductase (P5CR) to synthesize proline (Box 1; Fig. 1). This proline could then be transferred back into the mitochondria and again serve as a substrate for ProDH. There is support for this idea in the mammalian literature where it is proposed that such a cycle could serve to amplify proline-dependent ROS production in mitochondria (Tanner *et al.,* 2018; Phang, 2019). However, in plants, evidence for a P5C cycle is circumstantial and P5C produced by ProDH would need to cross both mitochondrial membranes to get to P5CR (via an unknown mechanism since no P5C transporter has been identified). It could be argued that the interaction of ProDH1 and P5CDH (and OAT) identified by Zheng *et al.* (2024) makes the existence of a P5C cycle less likely as it would sequester P5C away from transport to the cytosol. However, if such complexes are dynamic, their dissolution could release P5C and allow the P5C cycle to operate at appropriate times and places. One key to understanding the operation of the proline cycle versus the P5C cycle will be the identification of transporters that move proline and glutamate (and P5C?) into and out of the mitochondria (Box 1; Fig. 1). Activity of mitochondrial proline importer and proline–glutamate exchanger has been observed (Di Martino *et al.,* 2006), but the identity of genes encoding these activities is unknown. Basic Amino Acid Carrier 1 and 2 (BAC1 and BAC2) have been shown to act as mitochondrial transporters for ornithine and arginine (Hoyos *et al.,* 2003; Toka *et al.,* 2010), and BOU is a mitochondrial glutamate importer (Porcelli *et al.,* 2018). It is likely that other mitochondrial transporters also influence proline metabolism.

These questions are intertwined with the as yet unknown role(s) of post-translational modification and other protein interactions in controlling the activities of the proline metabolic enzymes. Alvarez *et al.* (2022) pointed out that both proline synthesis enzymes as well as transcription factors that regulate proline metabolism genes are putative phosphorylation targets of the TOR complex that monitors cellular energy status and controls many aspects of metabolism. Also, it was recently reported that activities of both ProDH and P5CDH were inhibited by direct physical interaction with Drought and Freezing Responsive Gene 1 (DFR1), a mitochondrial protein of otherwise unknown function (Ren *et al.,* 2018). Curiously though, it seems that Zheng *et al.* (2024) did not detect DFR1 in their analysis of ProDH1- and P5CDH-containing protein complexes. Questions have also been raised about the methods used to assay the DFR1 effect on ProDH activity (Alvarez *et al.,* 2022).

More broadly, the work by Zheng *et al.* (2024) reminds us that we still know surprising little about proline metabolism at the enzyme level. This lack of enzyme-level understanding persists despite the fact that proline is an integral part of stress resistance in many plant species and despite the fact that proline accumulation and stress regulation of proline metabolism genes, particularly *P5CS1*, are typically seen as canonical stress-related phenotypes used as markers of abiotic stress response. Zheng *et al.* (2024) have taken a step to fill the gap in understanding the enzymes of proline metabolism and how they operate together to match the activity of this important metabolic pathway to both the external environment and internal metabolic status of the plant.
